# Marginal resection as a potential curative treatment option of infantile fibrosarcoma with good response after chemotherapy: A case report of an ETV6-NTRK3 positive infantile fibrosacroma of the distal tibia

**DOI:** 10.1097/MD.0000000000034194

**Published:** 2023-07-07

**Authors:** Markus Weber, Frederik von Kunow, Axel Hillmann

**Affiliations:** a Faculty of Medicine, University of Regensburg, Regensburg, Germany; b Department of Orthopedic and Trauma Surgery, Barmherzige Brueder Regensburg Medical Center, Regensburg, Germany.

**Keywords:** infantile fibrosarcoma, marginal resection, surgery, therapy

## Abstract

The infantile fibrosarcoma (IFS) is a non-rhabdomyosarcoma soft tissue sarcoma with locally aggressive properties. State of the art therapy consists of neoadjuvant chemotherapy followed by wide resection according to the criteria of the musculoskeletal tumor society.

**Diagnoses:**

An ETV6-NTRK3 positive IFS of the distal tibia in a 21-months old child showed good response to chemotherapy.

**Interventions:**

Due to refusal of amputation marginal resection completing the margins with a high speed drill and filling the space with bone cement was performed.

**Outcomes:**

At latest follow-up 10 years after surgery, no recurrence was observed.

**Lessons:**

An individual therapy for surgical treatment of IIFS is recommended. This comprises marginal resection in instead of the golden standard “wide resection” in selected cases.

## 1. Introduction

The infantile fibrosarcoma (IFS) is a rare tumor entity affecting 5 in 1 million infants under the age of 1.^[1]^ It is classified as a non-rhabdomyosarcoma soft tissue sarcoma.^[2]^ This tumor presents with locally destructive properties. The most effected regions are the extremities, with more cases presenting at the lower extremities than at the upper extremities (66.4% vs 33.6%),^[3]^ followed by the trunk, the head, the region of the neck and the retroperitoneum.^[3–6]^ Metastases or the involvement of lymph nodes have been rarely observed. A high 5-year survival rate between 84% and 93% has been reported in literature under neoadjuvant systemic chemotherapy followed by wide resection of the tumor.^[2,7]^ Primarily, children under the age of 2 are affected with 30% to 50% of tumors detected at the time of birth and another 40% detected tumors preceding the child’s first birthday.^[3,8]^ Several tumors have also been detected intrauterine.^[1]^ It is the most common soft tissue sarcoma in this aging group. Histopathologically, IFS show dense cell spindle cells with a high proliferation rate as well as zones of necrosis. These are arranged in overlapping bundles with a herringbone pattern. The tumor cells have a fibroblastic and myofibroblastic ultrastructure and a nonspecific immune phenotype with variable positivity for desmin and actin.^[9]^ A recurrent translocation t(12;15) (p13;q25) with the transcript ETV6-NTRK3 is observed in the tumor.^[10,11]^ The therapeutic significance of this transcript has not been completely understood, as it also occurs in other tumors (mama carcinoma, acute lymphocytic leukemia and high-grade gliomas).^[12]^ A significant activation of the receptor tyrosine kinase (PI3K-AKT, MAPK, and SRC activation) has also been observed.^[13]^ The commonly recommended therapy is wide surgical resection,^[14,15]^ often resulting in amputations.^[16]^ In order to prevent amputations, neoadjuvant systemic therapies have been established to reduce the tumor size preoperatively.^[17,18]^ In some cases a spontaneous regression has been observed in non-resectable tumors after neoadjuvant chemotherapy, obviating the need for further surgical treatment.^[14]^ Postoperatively after resection of the IFS, no adjuvant chemotherapy is currently recommended according to the guidelines of the European soft tissue sarcoma group. However, a close follow-up is recommended.^[19]^ Adjuvant radiotherapy is not relevant for this young collective of patients.

## 2. Case report

**Figure 1.**
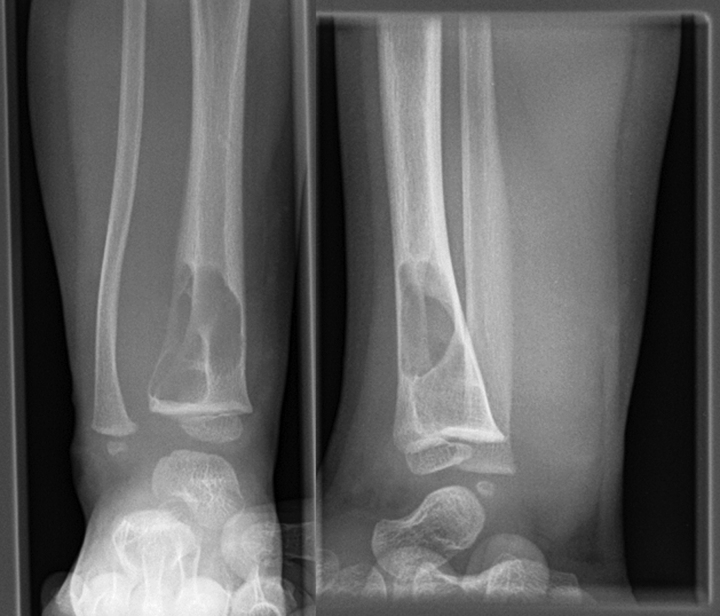
Plain radiographs show an osteolytic lesion of the distal tibial metaphysis.

**Figure 2.**
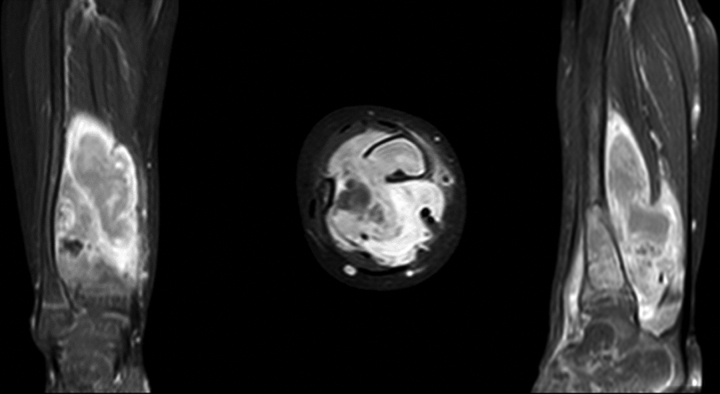
MRI (T1 with contrast) confirms the lesion in the distal tibia but reveals a huge soft tissue formation with consecutive bowing of the fibula with high contrast agent uptake. MRI = magnetic resonance imaging.

**Figure 3.**
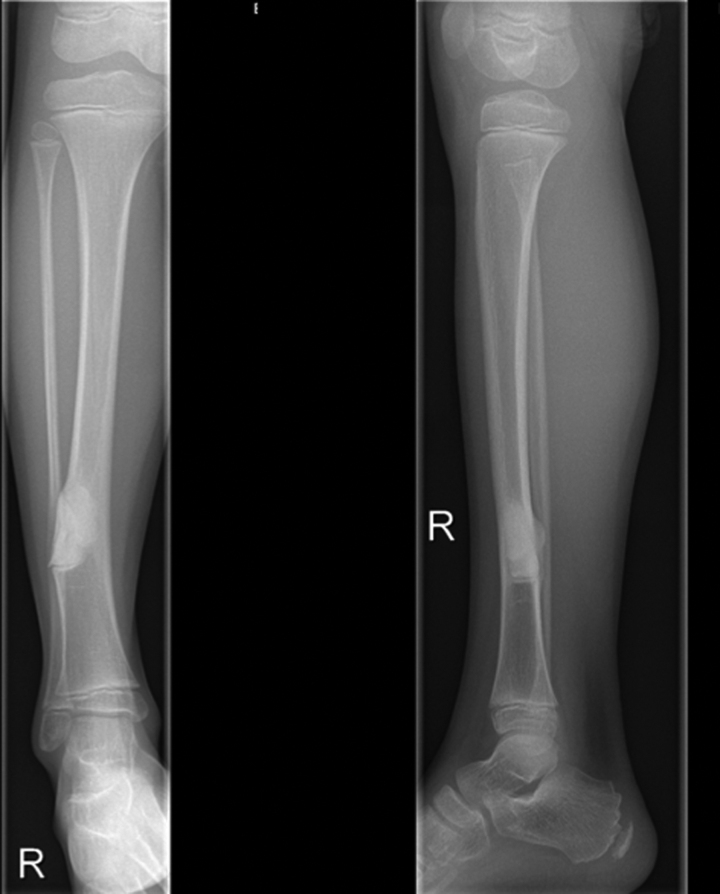
Radiographic image at the age of 9 showing the cement plombage after marginal resection of the tumor.

**Figure 4.**
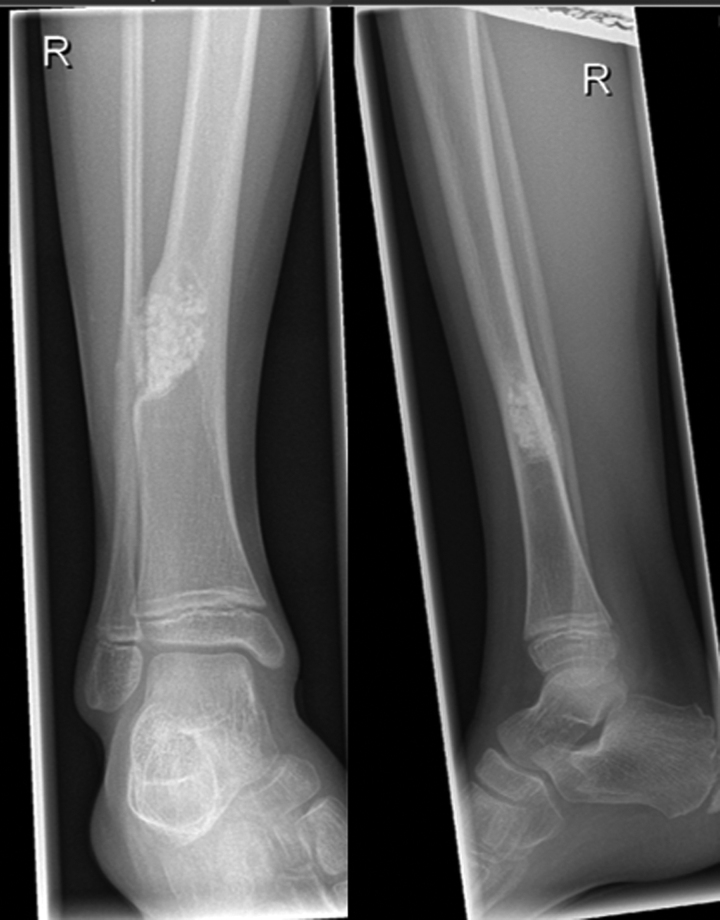
Radiographic image after removal of the cement plombage and filling with osteoconductive material at latest follow-up.

A 21-months old child presented with a swelling above the right ankle with consecutive increasing size. Initially, the patient had been referred to another hospital. No pain or muscular dysfunction was reported. The initial plain radiographic images showed an osteolytic lesion of distal metaphysis of the tibia consisting of several chambers (Fig. 1). A magnetic resonance imaging (MRI) scan with contrast agent obtained at the following day confirmed a tumor of the lower leg with the size of 2 × 3.5 cm including a significant soft tissue formation with consecutive bowing of the fibula (Fig. 2). The differential diagnosis assumed by the colleagues had been a malignant tumor or an aneurysmal bone cyst. The biopsy of the tumor had revealed the diagnosis of fibrosarcoma. Unfortunately, the biopsy had been taken using 2 different surgical approaches, one from the medial and one from the lateral side of the tibia. A staging computed tomography scan of the thorax and abdomen was obtained. No further metastasis or lymph node involvement were observed. The patient received neoadjuvant chemotherapy according to the Cooperative Weichteilsarkom Sudiengruppe-Guidance 2007 with vincristine, actinomycin D and cyclophosphamide (VAC). The first 3 cycles of the chemotherapy regime VAC were administered within 8 weeks and were well tolerated by the patient. After the last of the 3 cycles of chemotherapy a MRI-scan showed high regression of the tumor mass. Another three 3 cycles of chemotherapy regime VAC were performed within 8 weeks. Another MRI follow-up at the end of chemotherapy showed an additional discrete remission of the tumor mass. Due to the medial and lateral approach for biopsy, limb sparing surgery was impossible from a tumor surgical point of view to reveal tumor free margins and spare the surgical approaches in terms of a wide resection as recommended for malignant sarcoma. However, the parents strongly opposed amputation of the leg. Under these conditions a marginal resection of the tumor sparing the nerves and the blood vessels was performed. This included the application of a high-speed drill to complete the sclerotic margins of the bone. In addition, the bone was filled with a plombage of bone cement (Palacos®; Heraeus, Hanau, Germany) for 3 reasons: stabilization of the bone, anti-tumor effect of the heat reaction, easier radiographic follow-up (Fig. 3). During curing, the bone cement shows a thermal reaction with a heat development between 70°–90° Celcius. We decided against a protective osteosynthesis to prevent image artifacts for future MRI follow-ups. The patient was able to perform full weight bearing 2 weeks after the operation. The histopathology report of the tumor described a positive proof for Vimentin and negative proof for sm-actin, desmin, CD34, CD68, and pan-cytokeratin (CKMNF). The caldesmon-reaction was weakly positive. With the proliferation indicator Ki67 (MiB) max. 5% of the cell nuclei had responded positively. No mutation of the GNAS1-gene was detectable. A brake in the ETV – 6-gene (T-Nr. 14/001453) and an ETV6-NTRK3-fusion transcript with the for the infantile fibrosarcoma characteristic translocation t (12;15) was detected. Regular follow-ups were performed including MRI without any hint of tumor recurrence. Approximately 7 years later at the age of 9, revision surgery was performed. The cement plombage was removed and replaced with osteoconductive material. At the latest follow-up the tibial bone was consolidated and the patient without any complaints (Fig. 4).

## 3. Discussion

The clinical aspect of the IFS differs from the fibrosarcoma of the adulthood. The juvenile form seems to be less aggressive than in adults. A high survival rate and rare metastatic spread of 8% of cases have been reported in literature.^[6]^ The IFS mostly affects the distal portion of the extremities while the fibrosarcoma of the adulthood affects the femur.^[20]^ Because of the small number of medical cases no standard therapy has been established yet.^[3]^ Surgical treatment so far has consisted of a complete resection of the fibrosarcoma corresponding to an R0 resection including wide margins. For that reason, an amputation has been often inevitable. If a local excision is not possible because of the tumor size a regression of tumor mass should be tried by chemotherapy to reduce tumor size.^[17]^ The most frequently applied chemotherapy regime is vincristine, actinomycin D and cyclophosphamide (VAC). There are several studies describing a good clinical outcome despite marginal resection and local or metastatic recurrence.^[14,21–23]^ Sowash et al^[24]^ described in their article an IFS, which decreased in size due to neoadjuvant chemotherapy and thus became operable. Sait et al^[9]^ found 1 case of an IFS in a 5-month-old infant showing spontaneously a complete regression. The IFS was histologically confirmed by biopsy. In the course of 21 months no local recurrence was observed. This behavior has been described in 3 different case reports.^[25–27]^ The patient presented in this paper received a neoadjuvant chemotherapy followed by surgical marginal resection. The aim of the operation was to preserve the limb and prevent amputation which was opposed by the parents. The high speed drill and the heat development of the bone cement during curing might have supported to prevent from a local recurrence. The use of a cement filling additionally provides sufficient fracture prevention. Since no protection osteosynthesis was carried out it is possible to perform control MRI and safely exclude a local recurrence. This is essential because the rate of local recurrences is very high with 33%.^[7]^ Histological examination of the tumor showed the transcript ETV6-NTRK3. As described above, this has previously been detected in patients with spontaneous regression. After recurrence-free survival of 5 years, tumor cure could be assumed and thus removal of the cement plombage was considered. The biological restoration can either be performed using an autologous fibula graft or with a synthetic bone substitute. In this case, we chose osteoconductive synthetic bone due to the limited size of the osseous defect.

## 4. Conclusion

The IFS is a rare tumor entity. Neoadjuvant chemotherapy followed by resection with wide margins the standard therapy. In the current case we were able to show that if chemotherapy responds well, marginal resection of the tumor can be sufficient. High speed drilling and the heat development of the bone cement during curing seem to prevent local recurrence. The filling of the bone with bone cement prevents fractures and enables MRI scans postoperatively without metal artifacts in order to determine a possible recurrence of the tumor. Some case reports show a spontaneous remission of the tumor. The therapeutic significance of the transcript found in these patients has not yet been completely understood. In conclusion, it is not always necessary to resect the tumor with wide margins often resulting in amputation. Instead, an individual approach for this tumor entity is highly recommended evaluating the different therapy options. However, this is restricted to this tumor entity. In general or other malignant sarcoma in the childhood, wide resection remains the standard of care.

## Author contributions

**Conceptualization:** Markus Weber, Axel Hillmann.

**Data curation:** Markus Weber, Frederik von Kunow.

**Formal analysis:** Frederik von Kunow.

**Investigation:** Frederik von Kunow, Axel Hillmann.

**Methodology:** Frederik von Kunow.

**Project administration:** Markus Weber, Axel Hillmann.

**Resources:** Axel Hillmann.

**Supervision:** Axel Hillmann.

**Validation:** Axel Hillmann.

**Visualization:** Axel Hillmann.

**Writing – original draft:** Markus Weber, Frederik von Kunow.

**Writing – review & editing:** Markus Weber, Frederik von Kunow, Axel Hillmann.
